# Genome Sequence of the Multiple-Protease-Producing Strain *Geobacillus thermoleovorans* N7*,* a Thermophilic Bacterium Isolated from Paniphala Hot Spring, West Bengal, India

**DOI:** 10.1128/genomeA.01202-16

**Published:** 2016-10-27

**Authors:** Sucharita Bose, Trinetra Mukherjee, Urmimala Sen, Chayan Roy, Moidu Jameela Rameez, Wriddhiman Ghosh, Subhra Kanti Mukhopadhyay

**Affiliations:** a Department of Microbiology, The University of Burdwan, Burdwan, West Bengal, India; b Bose Institute, Centenary Campus, Department of Microbiology, Kolkata, West Bengal, India

## Abstract

Here, we present the draft genome sequence of *Geobacillus thermoleovorans* strain N7 (MCC 3175), isolated from Paniphala Hot Spring, West Bengal, India, which contains genes that encode several industrially and medically important thermostable enzymes like neutral protease, xylose isomerase, rhamnogalacturonan acetylesterase, nitrate and nitrite reductase, l-asparaginase, glutaminase, and RNase P*.*

## GENOME ANNOUNCEMENT

*Geobacillus thermoleovorans* strain N7 (MCC 3175) is a rod-shaped, Gram-positive, aerobic, thermophilic bacterium, isolated from Paniphala Hot Spring (63 ± 1°C, pH 7.6 ± 0.2), located at Barabani, Asansol, West Bengal (23°45′33″N, 86°58′54″E). This strain belongs to the family *Bacillaceae* under the phylum *Firmicutes*. The strain grew on a nutrient agar plate at (60 ± 1ºC) with an incubation period of 16 to 20 h.

The purified genomic DNA of N7 was sequenced on an Ion PGM sequencer (1) using a 318 chip. Raw reads totaling 14,977,65 bp were assembled using SPAdes version 3.8.0 into 226 contigs with a coverage of 52%. A genome comprising 3,400,891 bp was obtained, with the largest contig size being 1,88,606 bp and the smallest being 200 bp, with a GC content of 52.4%. The genome was annotated and analyzed using NCBI PGAP (http://www.ncbi.nlm.nih.gov/genome/annotation_prok), RAST server version 2.0 (2), and PATRIC (3). NCBI PGAP predicted 3,530 genes, with 3,403 coding sequences, 36 rRNA genes, and 86 tRNA genes with 5 noncoding RNA genes. Genes encoding for rhamnogalacturonan acetylesterase (EC 3.1.1.86), an enzyme responsible for deacetylation of the hairy region of pectin (4), and xylose isomerase (EC 5.3.1.5), which is capable of xylose and glucose isomerization, have been found. Assimilatory nitrate reductase (EC 1.7.99.4) and nitrite reductase (EC 1.7.1.4) convert nitrate to nitrite and finally to ammonia. Three kinds of protease, i.e., serine protease, metalloprotease, and neutral protease, are also present in N7. The genome sequence of *G. thermoleovorans* CCB_US3_UF5, closest member of N7 (99% identity) does not have rhamnogalacturonan acetylesterase and neutral protease (5). The bacterial neutral proteases (pH around 7) are very much in use in food industry because they generate less bitterness in hydrolyzed food proteins in comparison to animal proteases (6). Wet lab experiments have already been done for the confirmation of the enzymatic activity of these industrially important enzymes. Apart from that, several genes of medically useful enzymes have been found, namely, l-asparaginase, glutaminase, and RNase P. L-asparaginase and glutaminase have anticancer activity and are used to treat acute lymphoblastic leukemia (7). As reported, an engineered RNase P ribozyme variant has been used as a potential antiviral agent in reducing human cytomegalovirus gene expression and growth (8). Xenobiotic-degrading genes encoding for enzymes like catechol-2,3-dioxygenase (EC 1.13.11.2) and nitrilotriacetate monooxygenase (EC 1.14.14.10), which are responsible for the degradation of catechol and nitrilotriacetate, have been found. Genes for fructose and mannose metabolism, maltose and maltodextrin utilization, and fatty acid biosynthesis and degradation have also been found.

Genes with multiple resistance to some heavy metals like cobalt, cadmium, chromium, and mercury and antimicrobials like polymyxin and melittin, have been found, which were present in strain N7 but not in its closest neighbor *G. thermoleovorans* CCB_US3_UF5 (5). Genes for the uptake and biosynthesis of some compatible solutes like glycine, myo-inositol, l-proline, sarcosine, and trehalose were also found. Enzymes of the central metabolic pathways (i.e., glycolysis, TCA cycle, pentose phosphate pathway, glyoxylate cycle, and oxidative phosphorylation) were also found, which indicates the bacterium’s aerobic mode of respiration.

### Accession number(s).

This whole-genome shotgun project has been deposited at DDBJ/ENA/GenBank under the accession number MDCP00000000. The version described in this paper is the first version, MDCP01000000.
